# Effects of Microwave Treatment on Structure, Functional Properties and Antioxidant Activities of Germinated Tartary Buckwheat Protein

**DOI:** 10.3390/foods11101373

**Published:** 2022-05-10

**Authors:** Simeng Wang, Xianmeng Xu, Shunmin Wang, Junzhen Wang, Wenping Peng

**Affiliations:** 1 College of Biological and Food Engineering, Anhui Polytechnic University, Wuhu 241000, China; 15357201167@163.com (S.W.); xuxianmeng@sina.com (X.X.); pengwenping0501@163.com (W.P.); 2 Xichang Institute of Agricultural Science, Liangshan Yi People Autonomous Prefecture, Xichang 615000, China; wangjunzhen108@163.com

**Keywords:** microwave pretreatment, germination, Tartary buckwheat protein, functional properties, antioxidant activities

## Abstract

Tartary buckwheat protein (TBP) has promise as a potential source of novel natural nutrient plant protein ingredients. The modulating effects of microwave pretreatment at varying powers and times on the structure, functional properties, and antioxidant activities of germinated TBP were investigated. Compared with native germinated TBP, after microwave pretreatment, the content of free sulfhydryl groups in the germinated TBP increased, and the secondary structure changes showed a significant decrease in α-helix and an increase in random coil contents, and the intensity of the ultraviolet absorption peak increased (*p* < 0.05). In addition, microwave pretreatment significantly improved the solubility (24.37%), water-holding capacity (38.95%), emulsifying activity index (17.21%), emulsifying stability index (11.22%), foaming capacity (71.43%), and foaming stability (33.60%) of germinated TBP (*p* < 0.05), and the in vitro protein digestibility (5.56%) and antioxidant activities (DPPH (32.35%), ABTS (41.95%), and FRAP (41.46%)) of germinated TBP have also been improved. Among different treatment levels, a microwave level of 300 W/50 s gave the best results for the studied parameters. Specifically, microwave pretreatment could be a promising approach for modulating other germinated plant protein resources, as well as expanding the application of TBP.

## 1. Introduction

Tartary buckwheat (*Fagopyrum tataricum*) is an important medicinal and edible plant that is rich in bioactive substances such as protein, starch, and flavonoids, and has a high nutritional and medicinal value [1]. Tartary buckwheat protein (TBP) is the main bioactive substance of Tartary buckwheat, accounting for approximately 15~17% of the whole seed. It has a rich and balanced amino acid content and high nutritional value, and also has a variety of biological activities, such as lowering blood pressure, lowering blood sugar, and antioxidation [2,3,4]; as a high-quality natural plant protein, TBP has considerable development prospects.

The physicochemical and functional properties of protein affect the process, nutrition, and sensory elements of food to a great extent, and are important aspects to evaluate the potential of proteins and provide guidance for their application. Recently, various methods have been used to improve the structure and functional properties of proteins to achieve the purpose of improving food quality. As a natural biological treatment technology, germination has been widely used to improve the nutritional and processing characteristics of plants. During plant germination, the endogenous protease is activated, the protein is decomposed into amino acids and peptides [5], the protein molecular structure is unfolded, the polypeptide chain is broken, and the water/oil binding site is exposed, which is conducive to the improvement of functional properties such as solubility, foaming, and emulsifying properties [6]. Singh et al. [6] reported that germinated sorghum has better functional properties than native sorghum, and the solubility, emulsifying, and foaming properties of sorghum protein after germination at 25 °C for 48 h were 49.38%, 15.02%, and 185.71% higher than those of the control, respectively. Anti-nutritional factors in the plant, such as phytic acid and trypsin inhibitors, will reduce the sensitivity of protein and related enzymes, while germination treatment can inhibit or eliminate anti-nutrients in plant, so that the nutritional value, biological activity, and digestibility of protein can be improved to a certain extent [7,8]. Sharma et al. [8] showed that the protein digestibility of pigeon pea seeds was 69.86%, and its protein digestibility could be significantly increased to 98.27% after 48 h of germination.

At present, some new technologies have been developed to regulate the germination and growth of plants, including microwaves [9], ultrasonic [10], and electromagnetic waves [11]. As an environment-friendly and efficient processing technology, microwaves have become a research hotspot in the food industry. Microwave treatment has a special regulatory effect on the germination, growth, and enzymatic activity of plants; our previous research results showed that microwave treatment can promote the germination of Tartary buckwheat seeds, and the germination rate of Tartary buckwheat within 3–7 days after microwave treatment (300 W/75 s) was significantly higher than that of ultrasonic treatment [12]. After 300 W/50 s microwave pretreatment, the bud length and fresh weight of Tartary buckwheat after germination at 5 days significantly increased by 23.38% and 34.48% compared with the control, respectively [13]. The electromagnetic energy generated by microwaves can activate the activity of endogenous enzymes in seeds, promote the decomposition of proteins and the synthesis of amino acids in plants [9], and improve the in vitro digestibility and biological activity of proteins during the germination of plants [12,14]. Other studies have shown that microwaves, as a physical modification method, can safely and effectively improve the functional properties of proteins based on preserving plant nutrients [15]. Deng et al. [16] found that the protein digestibility of buckwheat increased by 0.8%, 3.6%, and 6.5% under high hydrostatic pressure (600 MPa, 60 °C, 30 min), microwaves (850 W/30 min), and boiling treatments, respectively: both high hydrostatic pressure and boiling treatment significantly reduced the content of free amino acids in buckwheat protein, whereas microwave treatment has little effect on it. Sun et al. [17] investigated the effects of soaking, grinding, ultrasound, and microwaves on the protein digestibility of pigeon pea flour. The results showed that only microwave treatment significantly increased its value from 54.4% to 71.6%. Therefore, using microwaves to stimulate plant germination can be an effective strategy to improve the content of bioactive metabolites, nutritional value, and processing quality of plants.

However, there are few studies on the protein structure, functional properties, and bioactivity of germinated plants after microwave pretreatment. Keeping in view the innovative approach of novel food processing, this study used Tartary buckwheat seeds as raw materials to study the effects of microwave pretreatment with a different power and treatment time on the molecular structure, functional properties (solubility, water-holding capacity, emulsifying activity index, emulsifying stability index, foaming capacity, and foaming stability), in vitro protein digestibility, and antioxidant activities (DPPH, ABTS, and FRAP) of germinated TBP in order to provide a theoretical and experimental basis for the development and utilization of functional TBP resources.

## 2. Materials and Methods

### 2.1. Materials

All the reagents (analytical grade) and standards were purchased from Sinopharm Chemical Reagent (Shanghai, China) or Sigma-Aldrich (Tokyo, Japan). Tartary buckwheat seeds were provided by Xichang Agricultural Science Research Institute (Xichang, China).

### 2.2. Preparation of Germinated Tartary Buckwheat Samples

Tartary buckwheat seeds with full grains, both pollution-free and defect-free, were cleaned and soaked in 1 g/L KMnO_4_ solution for 5 min, then washed with deionized water until clear, soaked with deionized water at room temperature for 4 h, where water was changed every 2 h, and finally placed in 50 °C water bathed for 15 min to accelerate germination.

Microwave pretreatment (Zhongwei Teaching and Research Instrument R&D, Wuhu, China): one group of Tartary buckwheat seeds was irradiated with microwave power of 300 W for 30, 50, 70, 90, and 110 s, respectively; the other group of seeds was irradiated with microwave power of 200, 300, 400, 500, and 600 W for 50 s, respectively. Each group containing approximately 50 seeds was evenly distributed in a petri dish for microwave treatment (after microwave irradiation of Tartary buckwheat seeds, the surface temperature of seeds was measured with a handheld infrared thermometer immediately). Control test: the group without microwave treatment (CK).

The control group and the Tartary buckwheat seeds after different microwave treatments were placed in a germination incubator (Heheng Instrument and Equipment, Shanghai, China) (80% RH, 25 °C) in the dark. Since the cultivation, the fresh Tartary buckwheat sprouts with uniform growth for 3, 5, and 7 days of germination were collected, respectively, and the fresh Tartary buckwheat sprouts were shelled, vacuum freeze-dried at minus 70 °C for 24 h (Beijing Songyuan Huaxing Technology Develop, Beijing, China), ground (IKA, Staufen, Germany), and stored in zip-lock PE bags at low temperature for later use.

**Figure 1:**
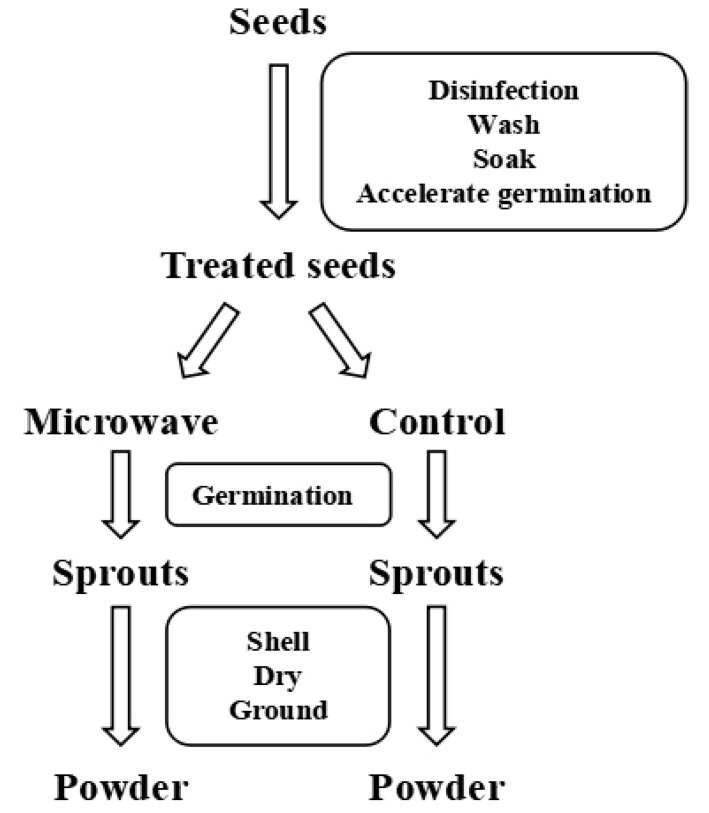
Flow chart of preparation of germinated Tartary buckwheat samples.

The flow chart of the preparation of germinated Tartary buckwheat samples is shown in Figure 1.

### 2.3. Preparation of TBP

TBP was extracted by the classic alkaline-dissolving and acid-precipitating method as described by Zhang et al. [18]. Tartary buckwheat powder and petroleum ether were mixed according to the material–liquid ratio of 1:5, stirred (Jie Rui Er Electric Appliance, Changzhou, China) continuously for 1 h, and the supernatant was removed after standing for 1 h. The precipitate was collected and dried in the fume hood. The precipitate was dispersed in deionized water to reach a concentration of solids of 10% (*w*/*v*), adjusted to pH 8.0 with 0.1 mol/L NaOH, stirred for 30 min, and centrifuged (Koki Holdings, Tokyo, Japan) (10,000 rpm, 10 min). The supernatant was collected, adjusted to pH 4.5 with 0.1 mol/L HCl, maintained at 4 °C for 4 h, and then centrifuged at 10,000 rpm for 10 min. The precipitate was collected, washed by centrifugation with deionized water 2 times, dispersed in deionized water and adjusted to pH 7.0, vacuum freeze-dried at minus 70 °C for 48 h, and stored for further analysis.

### 2.4. Determination of Free Sulfhydryl Content

The free sulfhydryl (SH_free_) of TBP was determined as previously reported [19]. One milliliter of 10 mg/mL protein solution (0.2 mol/L, pH 7.0 phosphate buffer solution) was dissolved in 4 mL of Tris-glycine buffer. The resulting solution was reacted with Ellman’s reagent (100:1, *v*/*v*) for 1 h at 25 °C and centrifuged at 10,000 rpm for 10 min, and the supernatant was collected and measured at 412 nm. The SH_free_ was determined as:

(1) $${\mathrm{SH}}_{\mathrm{free}}(\mathrm{nmol}/\mathrm{mg})=\frac{73.53\times A_{412}\times D}{C}$$

where *A*_412_ is the absorbance at 412 nm, *D* is the dilution factor, and *C* is the protein concentration (mg/mL).

### 2.5. Determination of Fourier Transform Infrared Spectroscopic

1–2 mg of dried TBP powder and an appropriate amount of KBr were mixed, ground, and compressed into a transparent sheet for Fourier transform infrared (FTIR) spectroscopy measurement (Shimadzu, Kyoto, Japan). The FTIR spectra were recorded from 4000 to 400 cm^−1^, with 20 scans at 2 cm^−1^ resolution.

### 2.6. Determination of Ultraviolet Spectra

With 0.01 mol/L, pH 7.0 phosphate buffer solution as a blank control, a protein solution with a concentration of 0.04 mg/mL was prepared, and ultraviolet (UV) (Shanghai Metash Instruments, Shanghai, China) spectra were collected at a wavelength of 200 nm to 400 nm.

### 2.7. Determination of Hydration Properties

The TBP powder was blended in phosphate buffer solution (0.2 mol/L, pH 7.0) to prepare a protein solution of 5 mg/mL. The solution was stirred for 30 min at room temperature and then centrifuged at 10,000 rpm for 10 min. The supernatant was assayed for protein by Coomassie brilliant blue method, and bovine serum protein was used as a standard substance to make a standard curve (y = 0.0057x + 0.0557, *R*^2^ = 0.9992). The total protein content was determined by the Kjeldahl method; refer to the method in GB 5009.5-2016. The solubility of protein was expressed as the percentage of supernatant protein content to total protein content.

The water-holding capacity (WHC) of protein was determined according to Tiwari et al. [20].

### 2.8. Determination of Emulsifying Properties

Emulsifying properties were determined according to Amza et al. [21] with slight modifications. Two milliliters of protein solution (10 mg/mL) was mixed with 1 mL of peanut oil, then homogenized (Dragon Laboratory Instruments, Beijing, China) at 18,000 rpm for 2 min. After homogenization, 50 μL of the emulsion from the bottom of the test tube was taken immediately and added with 5 mL of SDS (10 g/L). The absorbance of the solution was determined at 500 nm. The absorbance value was measured again at 10 min after homogenization according to the aforementioned method. The emulsifying activity index (EAI) and emulsifying stability index (ESI) were determined as:

(2) $${\mathrm{EAI}(m}^{2}/g)=\frac{2\times 2.303\times A_{500}\times DF}{C\times \rho \times \theta \times 10000}$$

(3) $$\mathrm{ESI}(\%)=\frac{EAI_{10}}{EAI}\times 100$$

where *A*_500_ is the initial absorbance, *DF* is the dilution factor (100), *C* is the concentration of protein (g/mL), *ρ* is the optical path (1 cm), *θ* is the proportion of the oil phase, and EAI_10_ is the EAI at 10 min after homogenization.

### 2.9. Determination of Foaming Properties

Foaming properties were determined according to Espinosa-Ramírez and Serna-Saldívar [22] with slight modification. A total of 10 mL of protein solution (10 mg/mL) was stirred for 2 min at high speed by a magnetic stirrer, and then quickly transferred to a graduated cylinder. The foam volume (*V*_0_) when the stirring stopped and the foam volume (*V*_30_) after 30 min were recorded. The foaming capacity (FC) and foaming stability (FS) were determined as:

(4) $$\mathrm{FC}(\%)=\frac{V_{0}-10}{10}\times 100$$

(5) $$\mathrm{FS}(\%)=\frac{V_{30}}{V_{0}}\times 100$$

### 2.10. Determination of In Vitro Protein Digestibility

The pepsin–trypsin in vitro digestion model was used for the determination of in vitro protein digestibility (IVPD). Tartary buckwheat powder was dissolved in HCl solution with pH 1.5 to form a solution of 100 g/L, stirred evenly by magnetic force, and heated to 37 °C; then, pepsin (P7000) was added in the ratio of enzyme to substrate of 1:100. After reacting at 37 °C for 2 h, the pH was adjusted to 7.0 with 1 mol/L NaOH to terminate the reaction, and then trypsin (P1750) was added in the ratio of 1:100, reacted at 37 °C for 2 h, and heated at 100 °C for 5 min to terminate the reaction. The IVPD was determined and calculated by the TCANSI method [23].

### 2.11. Determination of Antioxidant Activities

TBP was prepared with 0.2 mol/L, pH 7.0 phosphate buffer solution. The antioxidant activities were determined by referring to the method of Bian et al. [24] and Trolox was used as the standard curve. The linear regression equation of 1,1-diphenyl-2-picrylhydrazy (DPPH) radical scavenging capacity was y = 0.0841x + 4.1957 (*R*^2^ = 0.9995), the linear regression equation of 2,2′-azino-bis (3-ethylbenzothiazoline-6-sulfonic acid) (ABTS) radical scavenging capacity was y = 0.1854x + 6.2549 (*R*^2^ = 0.9992), and the linear regression equation of the ferric-reducing antioxidant power (FRAP) was y = 0.0012x − 0.0014 (*R*^2^ = 0.9999). The results were expressed in Trolox equivalent per gram of protein weight (μmol TE/g).

### 2.12. Statistical Analysis

All experimental results except FTIR and UV were observed as the mean value of three independent replicates, and the results were expressed as means ± standard deviations. Significant differences were analyzed with ANOVA using SPSS 22.0 Statistical software. *p* < 0.05 was considered statistical significance with Duncan’s test.

## 3. Results and Discussion

### 3.1. Effect of Microwave Treatment on SH_free_ Content of Germinated TBP

**Figure 2:**
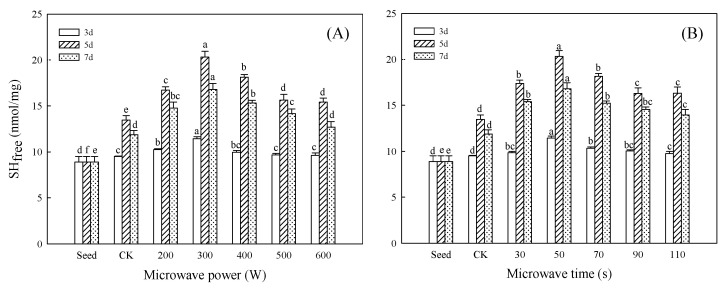
Effect of microwave treatment on SH_free_ content of germinated TBP. (**A**) Microwave time (50 s); (**B**) microwave power (300 W). Different lowercase letters indicate significant differences between the same germination time and different microwave treatment groups (*p* < 0.05).

The SH_free_ content of TBP is shown in Figure 2. Germination could increase the SH_free_ content of TBP, and the maximum of the CK group was 13.46 nmol/mg at 5 days of germination, which was 51.45% higher than that of seeds. The SH_free_ content increased significantly under different microwave powers and microwave times (*p* < 0.05), and reached the maximum value of 20.32 nmol/mg at 5 days after 300 W/50 s microwave pretreatment, which was 1.51 times that of CK. The content of SH_free_ plays an important role in protein structure. The changes in SH_free_ content represent the changes in protein structure and oxidation degree. The increase in SH_free_ content indicates the extension of the protein molecule, whereas the reverse indicates the folding of the protein molecule [25]. Studies have shown that germination can expand the protein molecular structure and break the polypeptide chain by activating protease activity in plants [6]. The change in SH_free_ content showed that microwave treatment could promote the proteolysis of germinated Tartary buckwheat, change the molecular conformation of the protein, and promote the expansion of the germinated TBP polypeptide chain and the exposure of internal SH_free_. In addition, microwave treatment could also promote the dissociation of protein subunits by protein hydrolase and break the disulfide bond to form new SH_free_ groups. The disulfide bond is a stable covalent side-chain naturally existing in protein, the chemical activity of the SH_free_ group is high, and the increase in its content is conducive to improving the functional properties of the protein [26]. Therefore, it is inferred that the protein molecules of Tartary buckwheat were extended after germination under microwave treatment, the protein stability decreased, and the functional properties increased. However, with the continuous increase in microwave power and treatment time, the content of the SH_free_ of germinated TBP decreased. Combined with Figure S1, it can be seen that the surface temperature of Tartary buckwheat seeds continued to rise with the increase in microwave power and treatment time. After 300 W/50 s microwave treatment, the surface temperature of Tartary buckwheat seeds was 34.87 °C, whereas the surface temperature of Tartary buckwheat seeds after 600 W/50 s and 300 W/110 s microwave treatment reached 55.97 °C and 51.63 °C, respectively. The decrease in the SH_free_ content of germinated TBP after microwave treatment may be due to the excessive heat of electromagnetic energy conversion generated by excessive microwave treatment, and the excessive temperature leads to the decrease or inactivation of enzyme protein activity in Tartary buckwheat seeds.

### 3.2. Effect of Microwave Treatment on Secondary Structure of Germinated TBP

The amide I region determined by FTIR spectroscopy is the characteristic vibration peak of the protein secondary structure. The region (1700–1600 cm^−1^) was fitted by PeakFit software to analyze the content of the α-helix and β-sheet, β-turn, and random coil structure, to analyze the changes in the secondary structure of the protein, of which, the α-helix and β-sheet are relatively stable ordered protein structures, and the β-turn and random coils are disordered structures [26,27].

**Figure 3:**
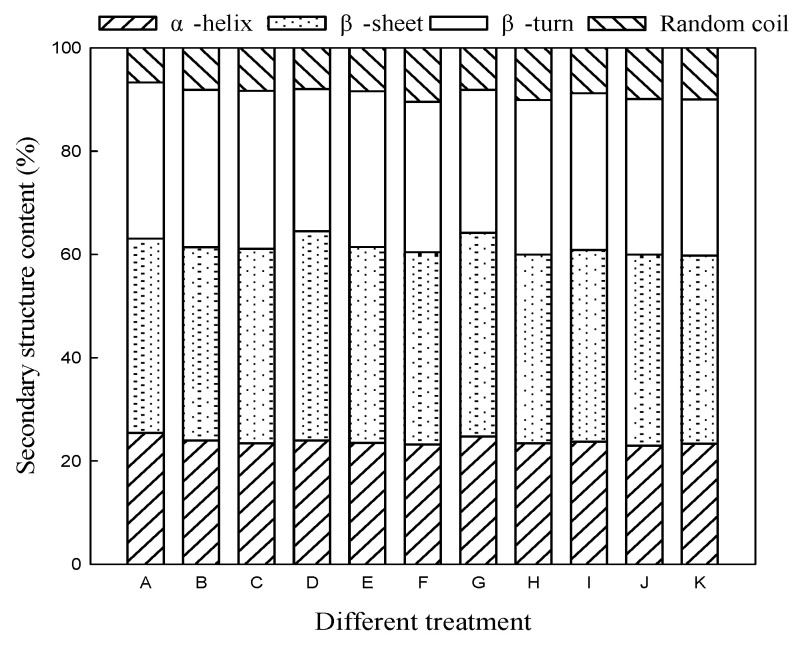
Effect of microwave treatment on secondary structure of germinated TBP. A: Seed; B: CK/3 d; C: CK/5 d; D: CK/7 d; E: 300 W 50 s/3 d; F: 300 W 50 s/5 d; G: 300 W 50 s/7 d; H: 200 W 50 s/5 d; I: 400 W 50 s/5 d; J: 300 W 30 s/5 d; K: 300 W 70 s/5 d.

It can be seen from Figure 3 that, compared with seeds, the relative content of the α-helix and β-sheet of TBP after germination decreased, and the relative content of the β-turn and random coil increased, whereas, in the later stage of germination, the proportion of ordered structure of TBP increased and the proportion of disordered structure decreased. The results showed that moderate germination would change the molecular structure stability of TBP and promote the unfolding of TBP molecules and the extension, rearrangement, and folding of peptide chains. Compared with CK, the relative contents of the α-helix and β-sheet of germinated TBP after microwave treatment decreased, whereas the relative contents of the β-turn and random coil increased, and the changes were more obvious with the increase in microwave power and treatment time. When the microwave power was 300 W and the treatment time was 50 s and germination for 5 days, the relative content of α-helix in TBP was the lowest (23.20%) and the relative content of the random coil reached the maximum (10.46%). Similar results were also reported by Sun et al. [17] when studying the effect of microwaves on the secondary structure of pigeon pea protein. It has been reported that disulfide bonds can facilitate the formation of β-sheet structures [28], so microwave treatment may reduce the relative content of β-sheet structures by promoting the breakage of disulfide bonds in germinated TBPs. Infrared spectroscopy analysis showed that moderate microwave pretreatment could change the secondary structure of germinated TBP, make its compact molecular structure looser, dissociate protein molecular subunits, stretch and unfold the partially ordered structure, and change the stability of the protein molecular structure. The functional properties of proteins are mainly affected by their spatial structure and intermolecular interactions. The tight and stable structure in proteins is not conducive to the exertion of protein functionality. The reduction in the relative content of ordered structures is beneficial to the improvement of protein functionality [29]. Microwave treatment can reduce the structural stability of TBP and make the protein structure more flexible, and it is more conducive to the contact between protein and the external interface, so it may be more conducive to the improvement of protein functional properties.

### 3.3. Effect of Microwave Treatment on UV Spectrum of Germinated TBP

**Figure 4:**
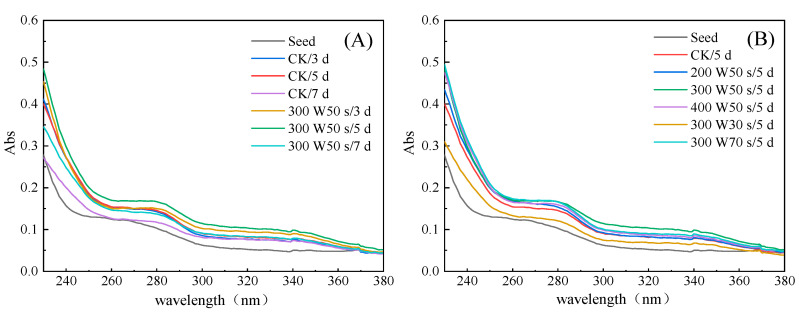
Effect of microwave treatment on UV spectrum of germinated TBP. (**A**) Different germination days; (**B**) Different microwave treatment.

As shown in Figure 4, the TBP after different treatments had the same shape of a UV absorption peak, and there was a relatively gentle peak near 280 nm, which was mainly dependent on the absorption of tyrosine, tryptophan, and phenylalanine [30].

It can be seen from Figure 4 that the absorption peak intensity of TBP near 280 nm increased after germination, the absorption peak reached the maximum at 5 days after germination, and the maximum absorption peak wavelength of TBP after germination was red-shifted compared with seeds, indicating that moderate germination can change the spatial conformation of TBP, expand protein molecules, expose chromogenic groups, and increase the intensity of absorption peaks. After microwave pretreatment, the UV absorption peak intensity of germinated TBP at 280 nm was greatly improved. With the increase in microwave power and treatment time, the UV absorption peak intensity first increased and then weakened, which was basically higher than that of CK. The intensity of the absorption peak reached the maximum when the microwave power was 300 W and the treatment time was 50 s for 5 days of germination. The experimental results showed that microwave treatment could promote greater changes in the spatial structure of TBP after germination. Under the action of protease, the interaction between protein molecules was destroyed and the exposure of more chromophores was increased to improve the intensity of the UV absorption peak of protein.

### 3.4. Effect of Microwave Treatment on Hydration Properties of Germinated TBP

**Figure 5:**
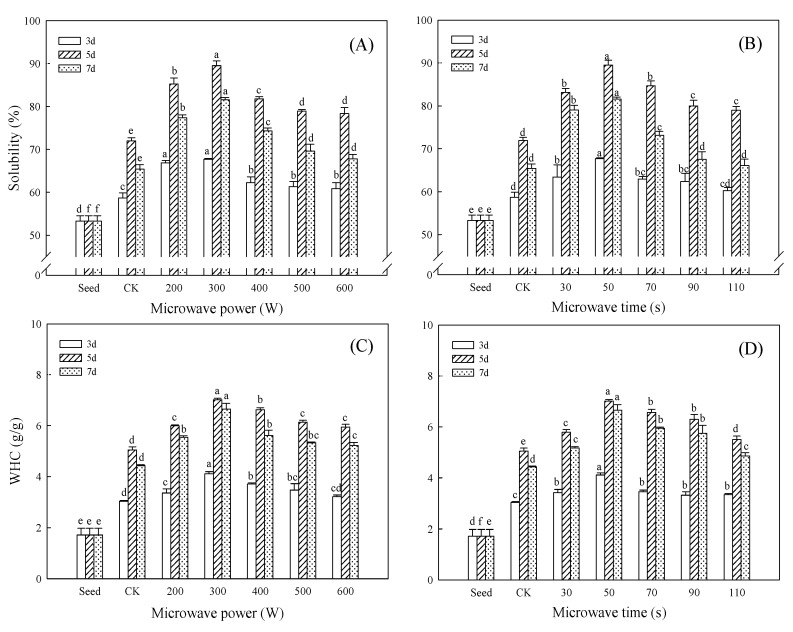
Effect of microwave treatment on hydration properties of germinated TBP. (**A**,**C**) microwave time (50 s); (**B**,**D**) microwave power (300 W). Different lowercase letters indicate significant differences between the same germination time and different microwave treatment groups (*p* < 0.05).

Solubility is the most important functional property of protein, and can measure the denaturation and aggregation of the protein and also determine and affect many functional properties, such as the emulsifying and foaming of protein [31]. There were differences in the effects of different treatments on the solubility of TBP. As shown in Figure 5A,B, the solubility of TBP increased first and then decreased with the extension of germination time, and the solubility of different treatment groups all reached the peak on the 5 days, in which, the solubility reached the maximum value (89.51%) when treated with 300 W microwave for 50 s, which was 24.37% higher than that of CK and 1.68 times that of seeds. Moderate germination could improve the solubility of TBP: after germination, under the action of endogenous protease, the molecular structure of TBP expands, the surface charge of the molecule increases, and the interaction with water molecules increases, resulting in the increase in solubility. The decrease in protein solubility in the later stage of germination may be due to the continuous hydrolysis exposing the hydrophobic groups inside the molecule, and the binding effect of protein and water molecules is weakened [6]. The results showed that microwave pretreatment could further improve the solubility of TBP within 7 days of germination. The analysis showed that microwave treatment could enhance the activity of the endogenous protease of Tartary buckwheat during germination, increase the free amino and carboxyl groups, and enhance the ion interaction, leading to a significant increase in the solubility of TBP (*p* < 0.05). However, as the microwave power and treatment time continued to increase, the electromagnetic energy absorbed by the seeds was converted into heat energy, and the excessive temperature reduced its promoting effect on the endogenous enzymes of Tartary buckwheat. A decreased protease activity limits the degree of protein hydrolysis and consequently decreased solubility.

WHC is the capacity of proteins to bind water and directly affects the texture, taste, and viscosity of food products [32]. Previous studies have proposed that the WHC of viscous foods was 1.49–4.72 g/g [33]; our results indicated that TBP germinated after microwave treatment could be an important ingredient in the food industry. As shown in Figure 5C,D, the WHC of TBP first increased and then decreased with the increase in germination time, and the WHC of TBP in the CK group reached 5.05 g/g at 5 days of germination. The WHC of TBP within 7 days of germination after microwave treatment was higher than CK, and the WHC of TBP was the highest (7.01 g/g) when the microwave level at 300 W/50 s and germinated for 5 days, which was 38.95% higher than that of CK. Compared with CK, the TBP germinated after microwave treatment showed significant differences (*p* < 0.05). Similar to previous studies, microwave treatment promoted the expansion of the molecular surface structure, and the loose network structure effectively improved the binding capacity between protein and water. The exposure of the water-binding sites on the polar group of the polypeptide side chain is prone to hydration, and the capillary action of the protein increases, which, in turn, leads to incremental increases in the WHC of the protein [34]. Almaiman et al. [15] reported that microwave-treated sorghum seeds at 350 W/45 s increased the WHC of protein from 3.30 mg/mL to 4.33 mg/mL, an increase of 31.21%, but slightly reduced protein solubility. In this study, microwave pretreatment can significantly improve the solubility of germinated TBP, and the WHC of TBP germinated for 5 days after 300 W/50 s microwave treatment was 4.08 times that of seeds. The results showed that germination after microwave pretreatment can significantly improve the hydration properties of plant protein, which is more conducive to the application of plant protein in food processing.

### 3.5. Effect of Microwave Treatment on Emulsifying Properties of Germinated TBP

TBP has amphoteric groups, so it has better emulsifying properties in the oil–water mixture. EAI and ESI represent the adsorption capacity of protein at the oil–water interface and the ability to maintain two-phase stability during the formation of emulsion, respectively. They are the most commonly used indicators to evaluate the emulsifying properties of food proteins. The improvement of emulsifying properties is conducive to improving the quality of food [33].

**Figure 6:**
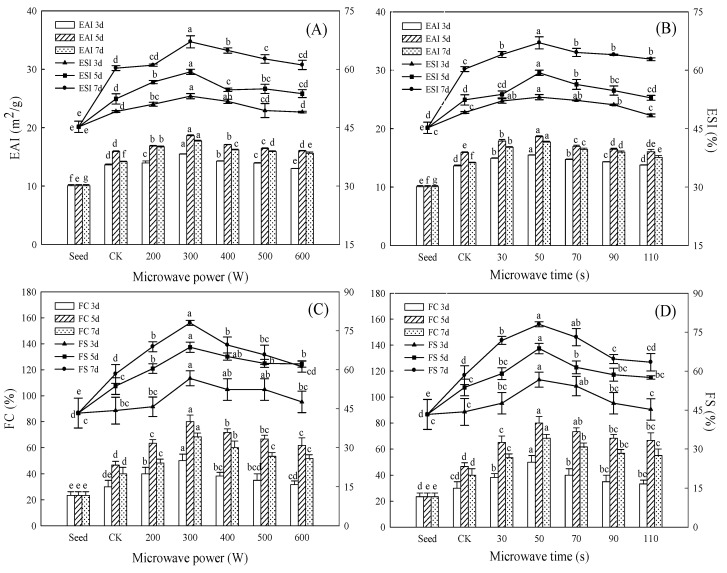
Effect of microwave treatment on emulsifying properties and foaming properties of germinated TBP. (**A**,**B**) Bar graph indicates EAI and line chart indicates ESI. (**C**,**D**) Bar graph indicates FC and line chart indicates FS. (**A**,**C**) microwave time (50 s); (**B**,**D**) microwave power (300 W). Different lowercase letters indicate significant differences between the same germination time and different microwave treatment groups (*p* < 0.05).

The emulsifying properties of TBP are shown in Figure 6A,B. The EAI first increased and then decreased with the extension of germination time, reaching the peak at 5 days of germination, whereas the ESI continued to rise with the extension of germination time and reached the maximum at 7 days of germination. Germination significantly improved the emulsifying properties of TBP (*p* < 0.05): germination will hydrolyze TBP molecules, and the hydrophilic and hydrophobic groups will diffuse to the oil–water interface, respectively, and interact with each other to improve the interface strength. Meanwhile, the EAI and ESI were improved to varying degrees. With the exposure of internal hydrophobic groups increasing, the water–oil balance will be broken and some polymers will be formed, and the EAI will decrease and the ESI will increase. Similar results were also reported by Singh et al. [6]. Germination can significantly improve the solubility of sorghum proteins. Proteins with a high solubility are more surface-active agents and promote good oil-in-water emulsion, which results in the better emulsifying properties of the protein, and the EAI and ESI of sorghum proteins after germination were increased by 15.02% and 18.62% compared with seeds, respectively. In addition, microwave pretreatment could further improve the emulsifying properties of TBP after germination, especially at 300 W/50 s microwave treatment, where the improvement of EAI and ESI after germination was the best, reaching 18.64 m^2^/g and 67.04% at 5 and 7 days of germination, respectively, which increased by 85.43% and 48.24% compared with seeds, and 1.17 and 1.11 times that of CK. Microwave treatment will make the protein structure of Tartary buckwheat looser after germination, promote the movement of protein to the oil–water interface, enhance the surface activity, reduce the interfacial tension, and loosen the polypeptide chain, and the change in protein conformation increases the surface-to-volume ratio, which is more conducive to adsorption at the oil–water interface. In addition, the protein molecules at the interface are more likely to form a viscoelastic film around the oil droplets to make the emulsion more stable [35], so the EAI and ESI were significantly improved; however, with the increase in microwave power and treatment time, the decrease in protease activity led to the decline in EAI and ESI.

### 3.6. Effect of Microwave Treatment on Foaming Properties of Germinated TBP

The foaming properties of the protein are usually expressed by FC and FS. Foaming properties are the most important functional properties of the protein, and provide a unique texture and taste for food and play an important role in the quality of food during food processing [36].

As shown in Figure 6C,D, the FC and FS of TBP both showed an upward trend in the early stage of germination; then, the FC of TBP began to decline in the later stage of germination whereas the FS still showed an upward trend. The FC and FS of germinated TBP after microwave treatment were significantly improved compared with CK (*p* < 0.05), and reached the maximum value (80.00%, 78.02%) at 5 and 7 days after germination after 300 W/50 s treatment, respectively, which was 1.71 times and 1.34 times higher than that of CK. During germination, TBP is hydrolyzed into a small molecular subunit structure, which has a better adsorption capacity at the water–air interface, and FC and FS increased. In the late stage of germination, the hydrophobic group is exposed, the flexibility of the molecule increases, the protein aggregates at the interface to form an elastic network structure to prevent the foam from coalescing, and the FC decreased and the FS increased, which is also consistent with the findings of a previous study [37]. Su [38] showed that microwave treatment can significantly improve the foaming properties of quinoa protein, and excessive microwave treatment resulted in the thermal deformation of protein and destroyed the foaming properties of protein. The changes in foaming properties of germinated TBP after microwave treatment showed that low-power and short-time microwave treatment could expand the molecular structure of germinated TBP, which is easy to quickly adsorb on the water–air interface, and the expansion of the peptide chain at the interface can form a relatively stable cross-linked network structure to wrap the air to stabilize the foam [39]. Therefore, both the FC and FS were remarkably enhanced.

### 3.7. Effect of Microwave Treatment on IVPD of Germinated TBP

**Figure 7:**
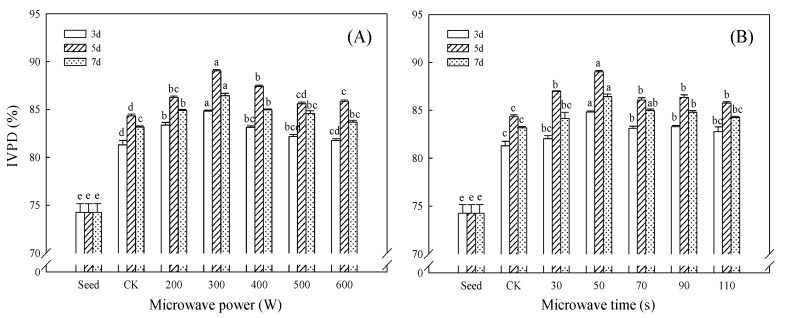
Effect of microwave treatment on IVPD of germinated TBP. (**A**) Microwave time (50 s); (**B**) microwave power (300 W). Different lowercase letters indicate significant differences between the same germination time and different microwave treatment groups (*p* < 0.05).

The IVPD is one of the main indicators to evaluate the protein nutritional value. The IVPD of TBP first increased and then decreased with the increase in germination time (Figure 7). During germination, various hydrolases are activated, storage proteins are modified and degraded into short polypeptides and free amino acids, and the protein structure and metabolism are transformed, making them more susceptible to enzymatic hydrolysis (pepsin and trypsin attack), thereby improving the IVPD [40]. Various anti-nutritional factors in Tartary buckwheat will form complexes with proteins to increase the degree of cross-linking and reduce the sensitivity of enzymes to protein attack. Studies have found that, under the action of protease, anti-nutritional factors such as trypsin inhibitors are reduced or completely hydrolyzed, which improves the IVPD [8]. The results showed that microwave treatment could significantly improve the IVPD of germinated TBP (*p* < 0.05). The IVPD of TBP was the highest when treated with 300 W microwave for 50 s and germinated for 5 days, which was 89.02%, 1.20 times that of seeds, and increased by 5.56% compared with CK. Moderate microwave treatment can promote the enhancement of the endogenous protease activity of germinated Tartary buckwheat, reduce the activity of anti-nutritional factors, and promote the hydrolysis of protein macromolecules into small molecules, providing higher surface areas and exposing more cleavage sites for the activity of digestive proteases. The protein easily interacts with pepsin/trypsin to significantly improve the IVPD. Other studies have shown that the secondary structure of the protein can also significantly affect its IVPD; for example, there is a negative linear correlation between the β-sheet and IVPD of soybean protein [17]. In this study, after microwave pretreatment, the ordered structure of germinated TBP decreased and the disordered structure increased (Figure 3), indicating that microwave treatment may also improve the IVPD by promoting the expansion and flexibility of the germinated TBP structure. Hassan et al. [14] showed that the IVPD of sorghum seeds increased from 71.31% to 74.70% after germination for 48 h after microwave treatment at 700 W/15 s, which was only 4.77% higher. Different treatment conditions and plant varieties will lead to differences in experimental results, the improvement of the IVPD of germinated TBP under moderate microwave treatment conditions is better, and it is easier to be digested and absorbed after being ingested by the human body. The improvement of the nutritional value of germinated TBP has increased its application and utilization in the plant protein food market.

### 3.8. Effect of Microwave Treatment on Antioxidant Activities of Germinated TBP

Organisms will constantly generate free radicals in the process of life. Free radicals have a strong oxidative effect. Excessive free radicals in the body can oxidatively denature nucleic acids, proteins, and other macromolecules, and change the cell structure and function. Oxidative stress caused by free radicals is one of the main factors of cell death and tissue damage. Excessive free radicals in organisms can cause aging, cardiovascular disease, and cancer if they are not removed in time [41]. Supplementing antioxidants can inhibit or eliminate free radicals in the body, reduce the oxidative damage caused to the human body, and play an important role in preventing diseases and maintaining health [42].

**Figure 8:**
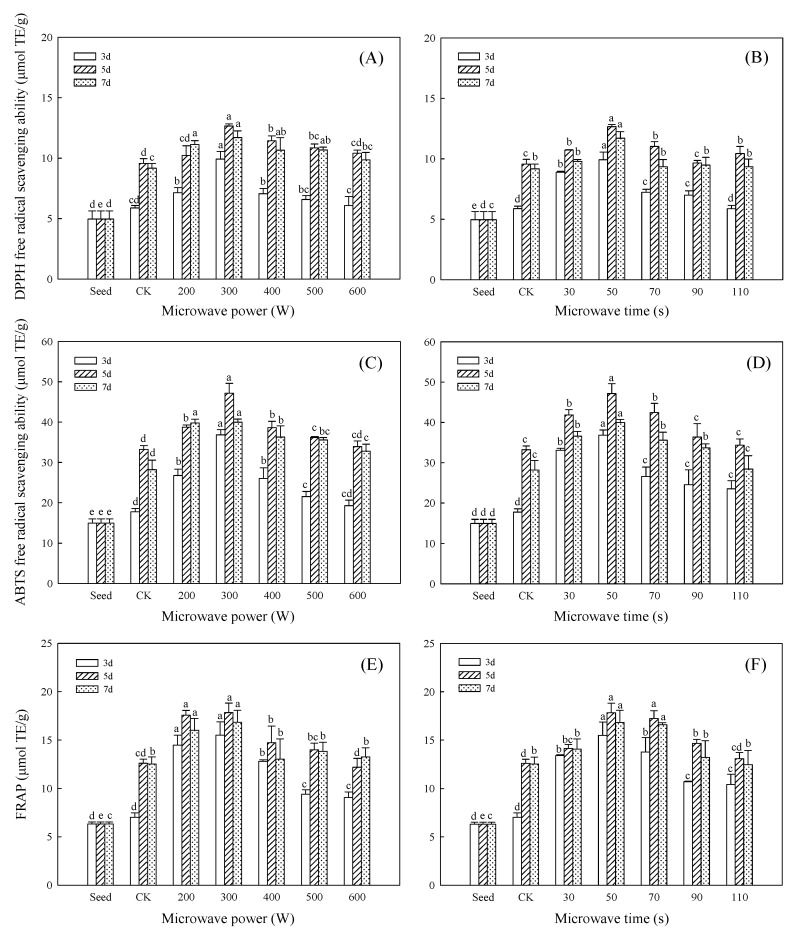
Effect of microwave treatment on antioxidant activities of germinated TBP. (**A**,**C**,**E**) Microwave time (50 s); (**B**,**D**,**F**) microwave power (300 W). Different lowercase letters indicate significant differences between the same germination time and different microwave treatment groups (*p* < 0.05).

The DPPH and ABTS methods are commonly used to determine the antiradical activity of tested compounds. Both systems accept and mix electron transfer and hydrogen transfer mechanisms, but they differ in the preferred deactivation mechanism: DPPH radicals are more susceptible to the latter, and ABTS to the former [43]. The FRAP method measures the ability of the tested compounds to reduce iron ions and reflects the total antioxidant capacity of the tested compounds. Thus, the application of these three methods will allow for a more comprehensive assessment of the antioxidant activity of the tested compounds. As shown in Figure 8, TBP had good antioxidant activities, and its antioxidant activities basically increased first and then decreased slowly with the increase in germination time. The antioxidant activities of TBP after germination for 5 days under 300 W/50 s microwave treatment were the strongest, and its scavenging capacity to DPPH and ABTS free radicals reached 12.65 μmol TE/g and 47.15 μmol TE/g, which were 32.35% and 41.95% higher than CK, respectively (Figure 8A–D). After microwave treatment at 200 W/50 s, 300 W/50 s, and 300 W/70 s, the FRAP of germinated TBP increased significantly; in particular, at 5 days of germination after microwave treatment at 300 W/50 s, the FRAP of germinated TBP reached the maximum value of 17.83 μmol TE/g, which was 41.46% higher than that of CK (Figure 8E,F). However, the FRAP of TBP after excessive microwave treatment, such as germination for 5 days after 600 W/50 s microwave treatment and germination for 7 days after 300 W/110 s microwave treatment, was lower than that in the CK group. The results showed that moderate microwave treatment could improve the antioxidant activities of TBP after germination (*p* < 0.05), whereas excessive microwave treatment had certain negative effects.

Tartary buckwheat storage protein can be hydrolyzed to separate polypeptides with stronger antioxidant activities than ascorbic acid, and some amino acid residues such as sulfhydryl and phenolic hydroxyl groups have a strong reducibility [44]. It has also been reported that germination treatment can hydrolyze soybean protein into smaller molecules, and the antioxidant activity of soybean protein is significantly enhanced [45]. Another study has shown that microwave treatment can promote protein hydrolysis and improve the biological activity of the protein; after microwave treatment, the DPPH and ABTS radical scavenging capacities of Chia seed are increased by 35.13% and 8.61%, respectively, compared with the control [46]. The further increase in the FRAP of germinated TBP after microwave treatment indicates that microwave treatment may further promote the extension of the molecular structure of germinated TBP, form more short peptides, and expose free sulfhydryl and phenolic hydroxyl groups with a strong reducing ability inside the molecules. As a result, the total antioxidant capacity of germinated TBP was increased. The abilities of germinated TBP to scavenge free radicals were significantly improved after microwave treatment, which may be due to the fact that exogenous stimulation can promote the hydrolysis of germinated TBP, protein structure stability decreases results in the flexibility of chains, the polypeptide chain is expanded and easily broken into smaller-sized polypeptides, and the separation or enrichment of electron-donating substances in the hydrolyzate can better contribute to the electron termination free radical chain reaction [47], whereas the excessive microwave treatment will reduce the seed viability and inhibit the activity of some proteases in Tartary buckwheat, resulting in the decline in antioxidant activities of some treatment groups, even lower than that of the CK group. The results were expressed in terms of Trolox equivalent per gram of protein (μmol TE/g); combined with the data analysis of the three indicators, it can be seen that the scavenging capacity of TBP to ABTS free radicals was higher than the other two antioxidants after different treatments, and, under the optimum microwave treatment conditions, the improvement degree of germinated TBP to the ABTS free radical was the best, and its value was 47.15 μmol TE/g, which was 3.15 times that of seeds and 41.95% higher than that of CK (Figure 8C,D). ABTS is a water-soluble free radical [48], TBP has the strongest scavenging ability to ABTS free radicals, and the germinated TBP after microwave treatment has the best improvement in the free-radical-scavenging ability of ABTS, indicating that Tartary buckwheat has better hydration properties, such as solubility and WHC, whereas microwave treatment can improve the hydration properties of germinated TBP, which is consistent with the results of the hydration property determination in this study (Figure 5).

## 4. Conclusions

This study was carried out to evaluate the effects of microwave pretreatment on the structure, functional properties, and antioxidant activities of germinated TBP. Microwave pretreatment could promote the unfolding of the protein molecular structure of TBP during the germination process. The content of free sulfhydryl groups increased significantly, the secondary structure of the protein changed from ordered to disordered, the spatial conformation of the protein changed, and the exposure of the chromophore led to an increase in the intensity of the ultraviolet absorption peak. The solubility, water-holding capacity, emulsifying properties, foaming properties, in vitro protein digestibility, and antioxidant activities of germinated TBP were significantly improved by microwave pretreatment, and the results showed that the microwave treatment at 300 W/50 s was most beneficial to the improvement in the protein quality of Tartary buckwheat. Therefore, this study shows that low-power and short-term microwave pretreatment can be used as an effective strategy to improve the functional properties, nutritional value, and biological activity of germinated TBP, which can provide a certain reference for the development and application of functional TBP food.

## Data Availability

Not applicable.

## Supplementary Materials

The following supporting information can be downloaded at: https://www.mdpi.com/article/10.3390/foods11101373/s1, Figure S1: Effect of microwave treatment on surface temperature of Tartary buckwheat seeds.
