# Supplementary material for: Effects of Microwave Treatment on Structure, Functional Properties and Antioxidant Activities of Germinated Tartary Buckwheat Protein
Source: Foods. 2022 May 10;11(10):1373. doi: 10.3390/foods11101373 (PMC9142102; doi:10.3390/foods11101373)
Supplement: Supplementary file 1 [file foods-11-01373-s001.zip › foods-1701427-supplementary.pdf]

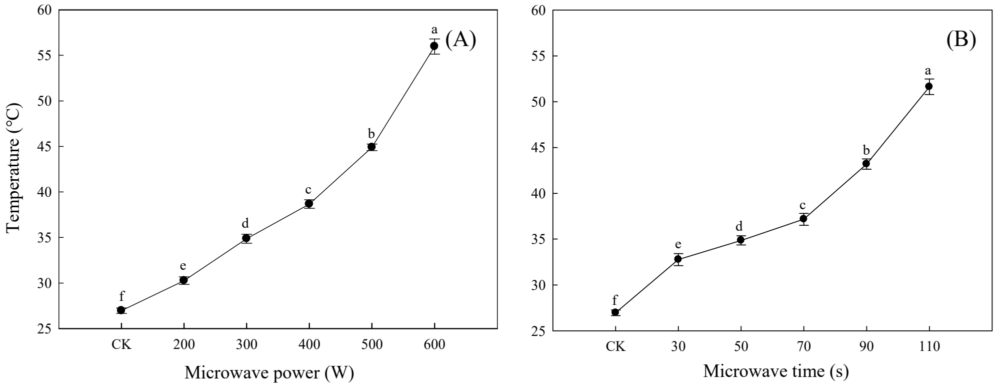

Figure S1. Effect of microwave treatment on surface temperature of Tartary buckwheat seeds. (**A**) microwave time (50 s); (**B**) microwave power (300 W). Different lowercase letters indicate significant differences between the different microwave treatment groups ($p < 0.05$).
